# Transgenic Expression of a Mutant Ribonuclease Regnase-1 in T Cells Disturbs T Cell Development and Functions

**DOI:** 10.3389/fimmu.2021.682220

**Published:** 2021-07-08

**Authors:** Gangcheng Kong, Yaling Dou, Xiang Xiao, Yixuan Wang, Yingzi Ming, Xian C. Li

**Affiliations:** ^1^ Immunobiology & Transplant Science Center, Houston Methodist Hospital, Texas Medical Center, Houston, TX, United States; ^2^ Transplantation Center of the 3rd Xiangya Hospital, Central South University, Changsha, China; ^3^ Department of Surgery, Weill Cornell College of Cornell University, New York, NY, United States

**Keywords:** Regnase-1, T cell development, positive selection, lymphopenia, skin transplantation, tolerance

## Abstract

Regnase-1 is an RNA-binding protein with ribonuclease activities, and once induced it controls diverse immune responses by degrading mRNAs that encode inflammatory cytokines and costimulatory molecules, thus exerting potent anti-inflammatory functions. However, Regnase-1 is extremely sensitive to degradation by proteases and therefore short-lived. Here, we constructed a mutant Regnase-1 that is resistant to degradation and expressed this mutant *in vivo* as a transgene specifically in T cells. We found that the mutant Regnase-1 transgenic mice exhibited profound lymphopenia in the periphery despite grossly normal spleen and lymph nodes, and spontaneously accepted skin allografts without any treatment. Mechanistic studies showed that in the transgenic mice thymic T cell development was disrupted, such that most of the developing thymocytes were arrested at the double positive stage, with few mature CD4^+^ and CD8^+^ T cells in the thymus and periphery. Our findings suggest that interfering with the dynamic Regnase-1 expression in T cells disrupts T cell development and functions and further studies are warranted to uncover the mechanisms involved.

## Introduction

Regnase-1 (also known as Zc3h12a and MCPIP1) is an RNase that is widely expressed in various immune cells, including macrophages, B cells and T cells (1). In the immune system, most of the mRNAs encoding cytokines and costimulatory molecules are short lived, which constitutes an important negative regulatory mechanism of immune responses (2, 3). Regnase-1 degrades mRNAs by recognizing a specific stem loop structure at 3’UTR of mRNA in concert with the helicase UPF1 (4, 5). Thus, Regnase-1 serves as an anti-inflammatory role by breaking down mRNAs that encode potent inflammatory cytokines, and consequently inhibits immune activation. Some of the known targets of Regnase-1 include mRNAs for *Il2, Il6*, *Il12b*, *c-Rel* and *Ox40* (5). As the immune responses are often tightly controlled in order to prevent collateral damage due to sustained immune responses (6, 7), Regnase-1 acts as an important feedback regulatory mechanism to negatively control immune responses.

However, Regnase-1 is also short lived *in vivo* and often degraded rapidly after immune activation (2, 8). For example, T cell antigen receptor (TCR) stimulation leads to the induction CARMA1-Bcl10-Malt1 signaling complex (CBM complex) in T cells, which is critical in mediating T cell activation and effector differentiation, primarily by activating the NF-κB and MAPK pathways (9–11). Importantly, activation of the Malt1 complex also cleaves the Regnase-1 (8, 12), which allows the prolonged expression of survival and key signaling molecules in activated T cells. Specifically, Malt1, which has proteolytic activities, cleaves Regnase-1 at the Arginine 111 site, leading to inactivation of Regnase-1 (8). On the other hand, TCR signaling can also upregulate the expression of Regnase-1, which is important in limiting persistent T cell activation (8). Thus, Regnase-1 plays a dynamic role in fine-tuning the activation of T cells.

In addition to activating T cells, signals transduced by TCR are critical for T cell development in the thymus (13). In developing thymocytes, the interactions between TCR and peptide-MHC complex trigger dynamic changes of gene expression in thymocytes in supporting cell survival and further maturation (14, 15). In fact, only after productive rearrangement of *TCR-β* gene and signaling *via* the pre-TCR can thymocytes progress forward beyond the DN3 stage (16, 17). In fact, recognition of the peptide-MHC complex on thymic stromal cells by the αβ TCR on developing thymocytes is vital for T cell survival and differentiation from DP to mature SP stage (18). The affinity of the interaction of the TCR and peptide-MHC complex determines thymocytes fate decisions. Weak interactions protect thymocytes from apoptotic death and promote the positive selection (19). Only a small proportion of DP thymocytes with functional TCR and proper affinity for the MHC complex can survive from thymic selection, and the majority of thymocytes with high affinity for the MHC complex and therefore strong TCR signaling undergo apoptosis (20).

In our study, we constructed a mutant Regnase-1, in which the arginine 111 was replaced with alanine (i.e., R111A), and expressed this mutant in T cells as a transgene to study how Regnase-1 affects TCR signaling, T cell development and functions. We found that this mutant mouse had profound lymphopenia in the periphery due to a developmental defect in the thymus. In a skin transplant model, we observed long term skin allograft survival in the mutant mice without any treatment. Our results highlight the importance of dynamic regulation of Regnase-1 in T cell activities and further suggest that Regnase-1 may be targeted to modulate T cell functions.

## Materials And Methods

### Mice

To create the R111A mutant transgenic mice, we inserted the CAG-LoxP-STOP-LoxP-Mcpip1(R111A)-P2A-EGFP cassette into the mouse *Rosa26* locus (21). *Cd4*-Cre, BALB/c mice were purchased from Jackson Laboratory (Bar Harbor, MA). *Mcpip1(R111A)* knock-in mice were crossed to *Cd4*-Cre mice to conditionally overexpress Regnase-1 in CD4^+^ T cells (Reg-1^CD4KI^). *Mcpip1* ^KI^ *Cd4*-WT mice were considered as WT littermate control. Mice at age of 6-8 weeks were used for all the experiments. All animals were maintained in specific pathogen free facility. All animal experiments in this study were approved by the Houston Methodist Animal Care Committee, in accordance with institutional animal care and use committee guidelines.

### Cell Line

HEK293 cells were obtained from the ATCC and were maintained in DMEM supplemented with 10% FBS and 1% penicillin streptomycin.

### Antibodies

Fluorescein conjugated antibody to mouse CD45 (Clone: 30-F11), CD3 (Clone: 17A2), CD4 (Clone: GK1.5), CD8 (Clone: 53-6.7), CD44 (Clone: IM7), CD25 (Clone: PC61), TCR-β (Clone: H57-597), CD69 (Clone: H1.2F3), CD62L (Clone: MEL-14), TNF-α (Clone: MP6-XT22), IFN-γ (Clone: XMG1.2), OX40 (Clone: OX-86), KLRG1 (Clone: 2F1/KLRG1) from BioLegend; CD5 (Clone: 53-7.3), Bcl-2 (Clone: 10C4), ICOS (Clone: 7E.17G9), PD-1 (Clone: J43), Foxp3 (Clone: FJK-16s) from Thermo Fisher were used in this study. Antibody for immunoblot in this study included the following: anti-Flag (F7425, Sigma), c-Myc antibody (9E10, Santa Cruz), anti-β-actin (C4, Santa Cruz), anti-Mcpip1 (604421, R&D), anti-Malt1 (cat #2494, Cell Signaling Technology).

### Mouse Skin Transplantation and Histology Staining

BALB/c tail skin allografts were transplanted onto the fully MHC-mismatched WT B6 or Reg-1^CD4KI^ recipients as previously described (22). More than 80% necrosis of the donor skin tissue was considered as rejection. Hematoxylin & eosin (H&E) staining was performed on paraffin sections of thymus and skin grafts.

### Flow Cytometry

The thymus and spleen were harvested to obtain single lymphocytes suspension. Red blood cells were lysed with ACK Lysing Buffer (Gibco). Live cell numbers were counted and the obtained samples (1×10^6^ cells) were then stained with fluorescein conjugated antibody in 100 µl MACS buffer (Miltenyi Biotec) for 30 min at 4°C in the dark for surface staining. For intracellular staining, total thymocytes were fixed and permeabilized using the Foxp3/Transcription factor staining kit according to the procedure recommended by ThermoFisher. For cytokine staining, splenocytes were stimulated for 5 h with phorbol 12-myristate 13-acetate (50 ng/ml) and ionomycin (500 ng/ml; Sigma-Aldrich) in the presence of GolgiStop (BD PharMingen). After surface marker staining, splenocytes were fixed and permeabilized with Cytofix/Cytoperm solution (BD PharMingen), and then stained with fluorescein conjugated TNF-α and IFN-γ antibody following the manufacturers’ instruction. All samples were acquired with LSRII (Beckton Dickinson) and the data were analyzed with FlowJo v10 software (Tree Star).

### Cell Sorting

Single thymocytes suspension was obtained as above. CD4^+^CD8^+^ DP thymocytes were sorted by an FACSAria cell sorter (BD Biosciences).

### RNA Isolation, cDNA Synthesis and RT-qPCR

Total RNAs from sorted DP thymocytes were prepared using Trizol reagent (ThermoFisher). The supernatants containing total RNAs were further purified with Direct-zol RNA MicroPrep Kit (Zymo Research). 0.1-1 µg of total RNAs were then reverse transcribed with iScript Reverse Transcription Supermix (BIO-RAD) according to the manufacturers’ instruction. The obtained cDNAs were tenfold-diluted and subjected into RT-qPCR experiments by using CFX96 Touch Real-Time PCR Detection System (BIO-RAD) and SsoAdvanced Universal SYBR Green Supermix (BIO-RAD). Primers for RT-qPCR were synthesized by Integrated DNA Technology (**Supplementary Table 1**). Expression was normalized to HPRT. The data were analyzed using the delta-Ct method.

### Retrovirus-Mediated Gene Transfer

The cDNA fragments encoding mouse Regnase-1, Malt1, Bcl10 were amplified by PCR and further cloned into the pMYs-IRES-EGFP retroviral vector (Cell Biolabs). The Arginine at 111 of Regnase-1 was mutated to Alanine using Q5 Site-Directed Mutagenesis Kit (NEB) following the manufacturers’ instruction. For transfection of HEK293 cells, Transporter 5 Transfection Reagent (Polysciences) was used according to the manufacturers’ instruction.

### Immunoblot Analysis

Protein extracts from HEK293 cells and thymocytes were prepared by washing the cells with cold PBS and then lysed in Pierce IP lysis buffer (87787, ThermoFisher Scientific) containing proteases and phosphatase inhibitor cocktail (78440, ThermoFisher) for 10 min. After 15,000g centrifugation for 10 min, the supernatants were transferred to new Eppendorf tubes. Whole cell lysates were subjected to immunoblot assay using standard procedures.

### Luciferase Assay

HEK293 cells were transfected with luciferase reporter plasmid pmiGLO containing the 3’UTR of *Il6* or empty vector, together with expression plasmid for Regnase-1 or empty (mock) plasmid. After 24 hours cultivation, cells were lysed and relative luciferase activity in lysates was detected using Dual-Luciferase Reporter Assay system (Promega). The gene encoding Renilla luciferase on pmiGLO plasmid was used as an internal control.

### *Ex Vivo* Thymocytes Cell Death Assay

1×10^5^ total thymocytes were cultured with PMA (50 ng/ml) and ionomycin (500 ng/ml; Sigma-Aldrich) in 200 µl of RPMI1640 media (10% FBS, 1% penicillin streptomycin, 50 µM 2-mercaptoethanol) in a 96-well flat bottom plate for 4h. Viability was measured by Annexin V and 7-AAD staining using Annexin V FLUOS staining kit (Roche).

### *Ex Vivo* TCR Stimulation Assay

96-well plates were coated with anti-CD3ε (5 µg/ml; Clone: 145-2C11, eBioscience) for 2h at 37 °C. After 1 time-wash with 100 µL PBS, 1×10^5^ total thymocytes were cultured in 200 µl of RPMI1640 media (10% FBS, 1% penicillin streptomycin, 50 µM 2-mercaptoethanol) with anti-CD28 (1 µg/ml; Clone: 37.51, eBioscience) for 24h. Viability was measured by Annexin V and 7-AAD staining using Annexin V FLUOS staining kit (Roche). Total RNA from DP thymocytes was extracted after 3h TCR stimulation.

### Statistical Analysis

The unpaired two-tailed Student’s *t* test was used for comparison between two groups. One-way ANOVA was used to generate *P* value between multiple groups. The Log-rank test was used to determine the *P* value of skin-graft survival time. Data were represented as mean ± SD and analyzed with Prism version 8 (GraphPad Software). The *P* value < 0.05 was considered statistically significant, as shown in the figure legends.

## Results

### Enhanced Stability of the Regnase-1 Mutant Without Interference of Its Rnase Activity

**Figure 1:**
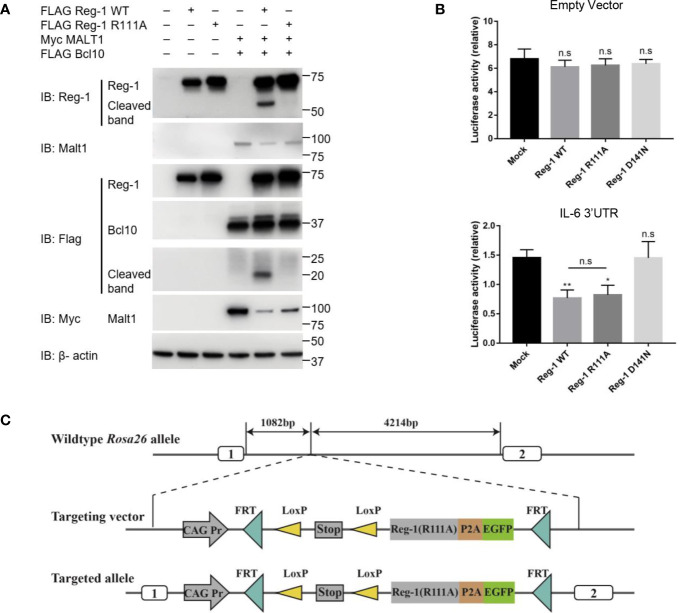
The effects of R111A mutant on Regnase-1 and the strategy of generating Regnase-1(R111A) transgenic mice. **(A)** Immunoblot analysis of lysates from HEK293 cells transfected with Flag-Regnase-1 (WT or R111A) and/or Myc-Malt1 and Flag-Bcl10 expression plasmids. **(B)** Relative luciferase activity of HEK293 cells transfected with pmiGLO plasmids and Regnase-1 expression plasmids. **(C)** Strategy for insertion of the R111A mutant Regnase-1 into the mouse *Rosa26* allele. Data are mean ± SD (n=3) from one experiment, representative of three independent experiments. n.s not significant; *P < 0.05; **P < 0.01 (one-way ANOVA test).

To investigate the stability and function of Regnase-1, we constructed a wild type and a mutant Regnase-1 where the arginine 111 was replaced by alanine (i.e., R111A). As shown in Fig 1A, co-transfection of wild type Regnase-1 with Malt1 and Bcl10 into HEK293 cells led to the cleavage of Regnase-1, resulting in a ~20 kDa and a ~53 kDa fragment. In contrast, the R111A mutant, when expressed in HEK293 cells, showed resistant to Malt1 mediated degradation (**Figure 1A**). We also performed luciferase assay to test the Rnase activity of the mutant Regnase-1. We transfected HEK293 cells with pmiGLO empty vector and Regnase-1 expressing plasmid. In the absence of the 3’UTR, luciferase activity of HEK293 cells was not influenced by the expression of Regnase-1. We then generated a luciferase reporter construct with 3’UTR of *Il6* mRNA, which has been shown to be a target of Regnase-1. Addition of *Il6* 3’UTR resulted in reduction of the luciferase activity in response to wild type and R111A mutant Regnase-1. In contrast, the overexpression of Rnase-inactive form (D141N) Regnase-1 failed to inhibit luciferase expression (**Figure 1B**) (8). Our results indicate that Rnase activity of Regnase-1 was not impacted by R111A mutant.

To further investigate the function of Regnase-1 in T cells, we generated the mutant Regnase-1 conditional knock-in mice (Reg-1^CD4KI^) by insertion of R111A mutant *Regnase-1* into *Rosa26* locus (**Figure 1C**) and crossed these mice with *Cd4*-cre mice (**Supplementary Figure 1A**). *EGFP* was the reporter gene to track cells expressing the mutant Regnase-1. The genotype of mouse was confirmed using genotyping (**Supplementary Figure 1B**). As compared to wild type B6 control mice, the Reg-1^CD4KI^ mice bred well, appeared normal, and born in accordance with Mendel’s fashion. Of note, we did not observe any non-lymphocytic abnormalities in the Reg-1^CD4KI^ mice.

### The Mutant Regnase-1 Transgenic Mice Exhibit Lymphopenia and Accept Skin Allografts

**Figure 2:**
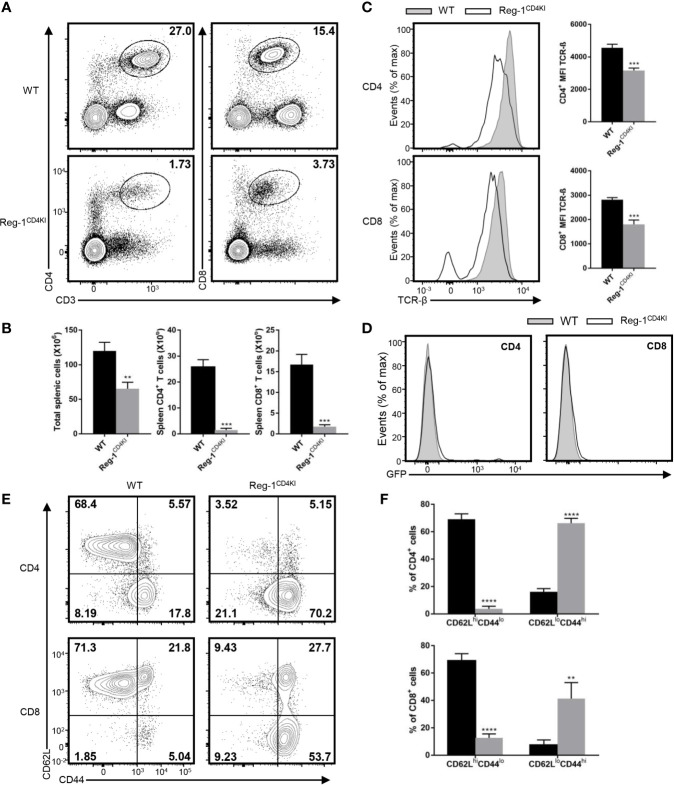
Phenotype of peripheral T cells in Reg-1^CD4KI^ mice. **(A)** Surface staining of CD3, CD4 and CD8 on WT or Reg-1^CD4KI^ splenocytes. The percentage of CD4^+^ and CD8^+^ T cell in CD45^+^ alive splenocytes. **(B)** The number of total splenocyte, CD4^+^ and CD8^+^ splenocytes from WT or Reg-1^CD4KI^ mice. **(C)** Flow cytometric analysis and mean fluorescence intensity of TCR-β on CD4^+^ or CD8^+^ splenocytes. **(D)** The analysis of EGFP expression in spleen cells from Reg-1^CD4KI^ mice or WT littermates at age of 6-8 weeks. **(E)** Expression of CD62L and CD44 on CD4^+^ or CD8^+^ splenocytes from WT or Reg-1^CD4KI^ mice. **(F)** The frequency of CD62L^hi^CD44^lo^ and CD62L^lo^CD44^hi^ subpopulations in the CD4^+^ or CD8^+^ cells. Data are mean ± SD (n=3) from one experiment, representative of two independent experiments. **P<0.01; ***P<0.001; ****P<0.0001 (unpaired two-tailed Student’s *t* test).

We examined the T cell compartment in peripheral tissues of Reg-1^CD4KI^ mice, total splenic cells, mature CD4^+^ and CD8^+^ T cells were both significantly decreased in spleen (**Figures 2A, B**), despite the normal size of the spleen (**Supplementary Figure 1C**). The number of Tregs in spleen was decreased, but the proportion of Tregs in splenic CD4^+^ T cell was comparable (**Supplementary Figure 2A**). Furthermore, the expression level of TCR-β on CD4^+^ or CD8^+^ T cell in spleen was also significantly lower in Reg-1^CD4KI^ mice, as compared to in WT mice (**Figure 2C**). And there were few mature T cells expressing EGFP (**Figure 2D**). Thus, in naïve mice, overexpressing Regnase-1 appeared to inhibit the maturation of T cells. In addition, peripheral CD4^+^ or CD8^+^ T cells from Reg-1^CD4KI^ mice had increased frequency of memory (CD44^hi^CD62L^lo^) cells than those in WT mice (**Figures 2E, F**), which was consistent with the observation that mice with T cell depletion therapies had more T cells with a memory phenotype.

**Figure 3:**
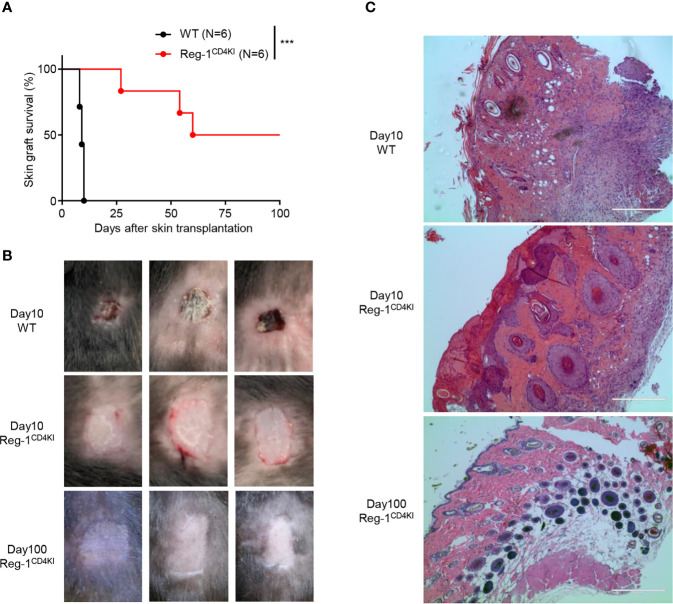
Regnase-1 promotes graft survival in skin transplantation model. **(A)** The percentage of skin allograft survival on WT or Reg-1^CD4KI^ recipients. ***P < 0.001; Log-rank test. **(B)** Representative images of BALB/c skin allografts at day 10 on WT recipients, and day 10 and 100 on Reg-1^CD4KI^ recipients. **(C)** Representative H&E staining images (×100) of BALB/c skin grafts at day 10 from WT recipients, and day 10 and day 100 from Reg-1^CD4KI^ recipients.

To investigate the T cell-mediated activities, we transplanted tail skins (0.8 cm × 0.8 cm) from BALB/c mice onto Reg-1^CD4KI^ and WT B6 recipients. The survival time of tail skin grafts on Reg-1^CD4KI^ recipients (mean survival time = 74.0 ± 12.9 days; n = 6) was significantly prolonged than that on WT recipients (mean survival time = 9.3 ± 0.3 days; n = 6) (**Figure 3A**). There were no signs of rejection of skin grafts on Reg-1^CD4KI^ recipients, but all skin grafts were rejected by WT recipients at day 10 (**Figure 3B**). In addition, the skin allografts from three Reg-1^CD4KI^ recipients survived more than 100 days (**Figures 3A, B**). H&E staining of skin grafts at day 10 showed that the skin grafts from WT recipients were destroyed and there were large number of lymphocytes infiltrating the graft tissue. In contrast, the skin grafts from Reg-1^CD4KI^ recipients at day 10 and day 100 were intact, with minimal infiltrating lymphocytes (**Figure 3C**). Taken together, overexpressing Regnase-1 in T cells led to the acceptance of allogeneic skin graft.

**Figure 4:**
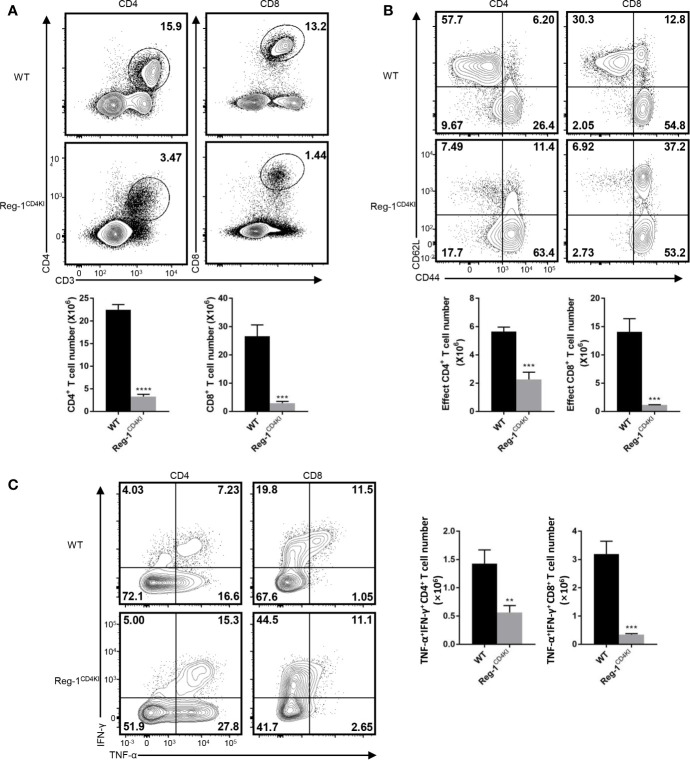
Reduced T effector cells in Reg-1^CD4KI^ recipient after skin transplantation. **(A)** The percentage and number of CD4^+^ or CD8^+^ T cells in spleen based on the expression of CD3, CD4 and CD8. **(B)** The percentage and number of effect T cells in CD4^+^ or CD8^+^ splenocytes, based on the expression of CD44 and CD62L. **(C)** The percentage and number of IFN-γ^+^TNF-α^hi^ T cells in CD4^+^ or CD8^+^ splenocytes. Data are mean ± SD (n=3) from one experiment, representative of three independent experiments. **P < 0.01; ***P < 0.001; ****P < 0.0001 (unpaired two-tailed Student’s *t* test).

We analyzed the phenotype of splenocytes at 10 days post-grafting. The number of CD4^+^ or CD8^+^ T cells were significantly lower in the spleen of Reg-1^CD4KI^ recipients (**Figure 4A**) as compared to that in WT mice. The number of activated CD4^+^ and CD8^+^ T cells was also decreased in Reg-1^CD4KI^ recipients, but a higher proportion of CD4^+^ T cells displayed an activated CD44^hi^CD62L^lo^ phenotype was observed (**Figure 4B**). In addition, there were no significant differences between the expression level of activation markers on CD44^hi^ effect T cells, such as ICOS, OX40, PD-1 and KLRG1 (**Supplementary Figure 2B**). We also assessed the effector functions of T cells by measuring intracellular expression of TNF-α and IFN-γ. As shown in **Figure 4C**, CD4^+^ and CD8^+^ T cells from Reg-1^CD4KI^ recipients producing TNF-α and IFN-γ decreased significantly, as compared to those from WT mice. With the stimulation of alloantigen, the clone expansion of effector T cells was inhibited by mutant Regnase-1. Taken together, Reg-1^CD4KI^ mice failed to reject allografts most likely due to lymphopenia and impaired T effector functions.

### Impaired T Cell Development in the Mutant Regnase-1 Transgenic Mice

**Figure 5:**
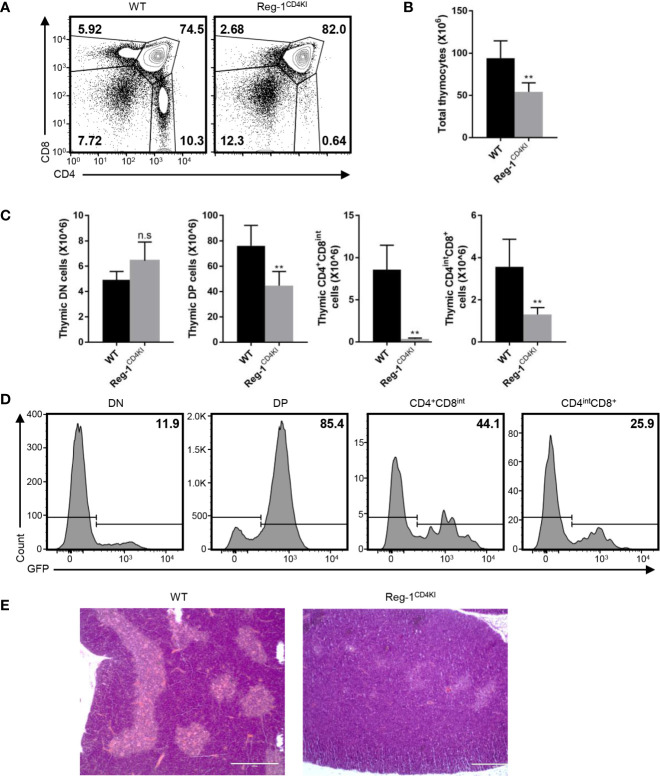
Defective T cell development in Reg-1^CD4KI^ mice. **(A)** Surface staining of CD4 and CD8 on WT or Reg-1^CD4KI^ thymocytes, gated on CD45^+^ alive cells. **(B)** Bar graphs representing the number of total thymocyte in WT or Reg-1^CD4KI^ mice. **(C)** Bar graphs representing the number of DN, DP, CD4^+^CD8^int^ or CD4^int^CD8^+^ thymocyte subpopulations. **(D)** The percentage of GFP^+^ cells in DN, DP, CD4^+^CD8^int^ or CD4^int^CD8^+^ thymocytes subpopulations in Reg-1^CD4KI^ mice. **(E)** Representative H&E staining images (×100) of thymus from WT or Reg-1^CD4KI^ mice. The cortex (darker) and medulla (lighter) can be distinguished by the intensity of staining. Data are mean ± SD (n=5) from one experiment, representative of three independent experiments. n.s not significant; **P < 0.01 (unpaired two-tailed Student’s *t* test).

Considering that the number of mature T cells reduced dramatically in Reg-1^CD4KI^ mice, we focused on the inhibitory effect of Regnase-1 on T cell development. In the thymus, very few CD4^+^CD8^int^ thymocytes were observed in Reg-1^CD4KI^ (**Figure 5A**), and the number of total thymocytes was significantly lower in Reg-1^CD4KI^ mice (**Figure 5B**). In addition, the numbers of DP, CD4^+^CD8^int^ and CD4^int^CD8^+^ thymocytes were also significantly decreased in Reg-1^CD4KI^ thymus, but the number of DN thymocytes were comparable (**Figure 5C**). According to the expression of EGFP, R111A mutant Regnase-1 started to express at DP stage (**Figure 5D**). During thymocytes maturation, T cells migrate from thymic cortex to thymic medulla. The architecture of the thymus was also altered in Reg-1^CD4KI^ mice, as characterized by much smaller areas of the medulla (**Figure 5E**). These observations suggested that Regnase-1 likely disrupts maturation of T cells in the thymus and led to fewer mature T cells in peripheral tissues.

### Impaired Positive Selection of Thymocytes in the Mutant Regnase-1 Transgenic Mice

**Figure 6:**
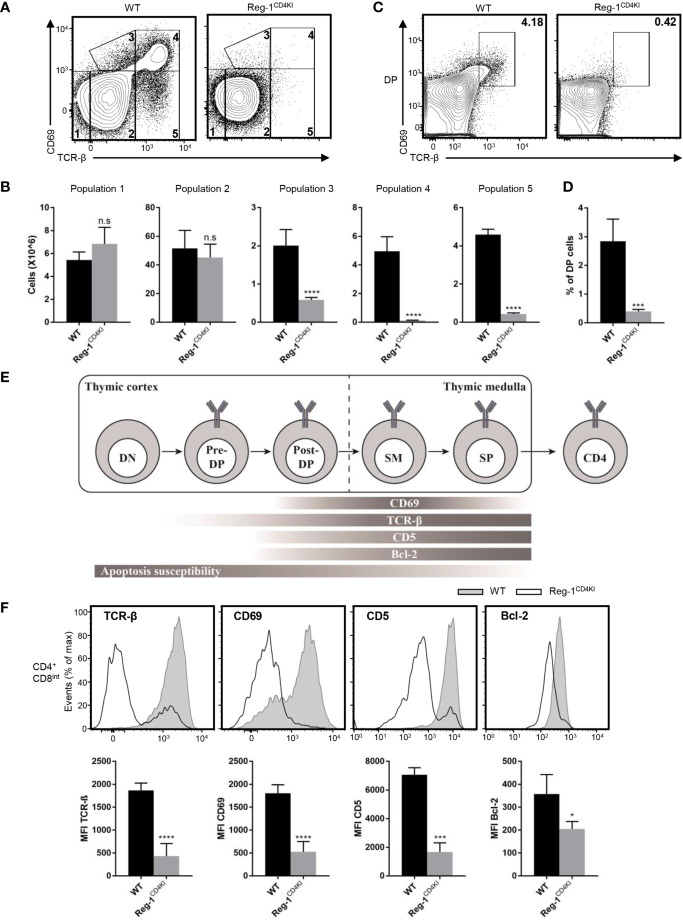
Mutant Regnase-1 impairs thymocyte positive selection *in vivo*. **(A)** Flow cytometry analysis of the surface expression of CD69 and TCR-β on WT or Reg-1^CD4KI^ thymocytes, gated on CD45^+^ alive cells. The number in outlined area indicates five subpopulations during thymocytes development. **(B)** Bar graphs representing the number of five subpopulations at different stage during T cell development in WT or Reg-1^CD4KI^ thymus. **(C)** Flow cytometric analysis of DP thymocytes through thymic positive selection based on the expression of CD69 and TCR-β, gated on CD45^+^ DP thymocytes. **(D)** Bar graph indicates the percentage of post-selection DP (CD69^+^TCR-β^hi^) thymocytes. **(E)** Schematic graph of CD4^+^ T cell development in thymus. T cells undergo differentiation from DN to DP to CD4 or CD8 single positive cell, accompanied with changes of several surface and intercellular markers (Pre-DP: pre-selection double positive cells; Post-DP: post-selection double positive cells; SM: susceptible to apoptosis mature cells). **(F)** Flow cytometric analysis and mean fluorescence intensity of TCR-β, CD69, CD5 and Bcl-2 in gated CD4^+^CD8^int^ thymocytes from WT or Reg-1^CD4KI^ mice. Data are mean ± SD (n=5) from one experiment, representative of three independent experiments. n.s not significant; *P < 0.05; ***P < 0.001; ****P < 0.0001 (unpaired two-tailed Student’s *t* test).

Next, we investigated the specific developmental stage at which thymocytes were blocked in Reg-1^CD4KI^ mice. The mutant Regnase-1 was not expressed during DN stage, hence had little influence on DN thymocytes. The development of thymocytes from DN1 to DN4 was normal in Reg-1^CD4KI^ mice (**Supplementary Figure 3A**). Then, we quantified cells at five different stages based on the expression level of TCR-β and the activation marker CD69 (23). We found that the proportion and the number of thymocytes in population 1 (TCR^lo^CD69^lo^) or population 2 (TCR^int^CD69^lo^) were comparable between Reg-1^CD4KI^ mice and their WT littermates. But Reg-1^CD4KI^ mice had significantly fewer cells in population 3 (TCR^int^CD69^hi^), population 4 (TCR^hi^CD69^hi^), and population 5 (TCR^hi^CD69^lo^) (**Figures 6A, B**). We further investigated the expression of CD4 and CD8 gated on each population. Thymocytes in population 1 were mostly DN and DP cells; in population 2 were most preselection DP cells; in population 3 were cells undergoing selection; in population 4 were post-positive-selection thymocytes; in population 5 were SP cells ready for migrating to the periphery (**Supplementary Figure 3B**) (23).

We stained surface and intracellular TCR-β separately and observed that the levels of total TCR-β on DP thymocytes were comparable between Reg-1^CD4KI^ mice and their WT control, but the surface expression of TCR-β was impaired on CD4 single positive cells in Reg-1^CD4KI^ mice (**Supplementary Figure 3C**). Immature thymocytes upregulate CD69 and TCR-β during the process of positive selection (13). We detected the expression of TCR-β and CD69 in thymocytes at DP stage. We found that the population of post-selection DP T cells (TCR^hi^CD69^hi^) were extremely fewer in Reg-1^CD4KI^ mice (**Figures 6C, D**). According to prior studies, we employed a staging scheme to show the changes of several markers during the process of T cell development (**Figure 6E**) (14, 15). CD5 can be used as an indicator for TCR signal strength during thymic development (13), and the anti-apoptotic factor Bcl-2 can be induced by TCR signal to promote the survival of developing thymocytes (14, 23). We analyzed the expression levels of TCR-β, CD69, CD5 and Bcl-2 in CD4^+^CD8^int^ thymocytes. Results showed that the expression of these markers were significantly lower in Reg-1^CD4KI^ mice (**Figure 6F**). These data indicated that TCR signaling during positive selection was disrupted by Regnase-1, which might contribute to the blockage of thymocytes during positive selection.

### Thymocytes That Express the Mutant Regnase-1 Are More Susceptible to Apoptosis

**Figure 7:**
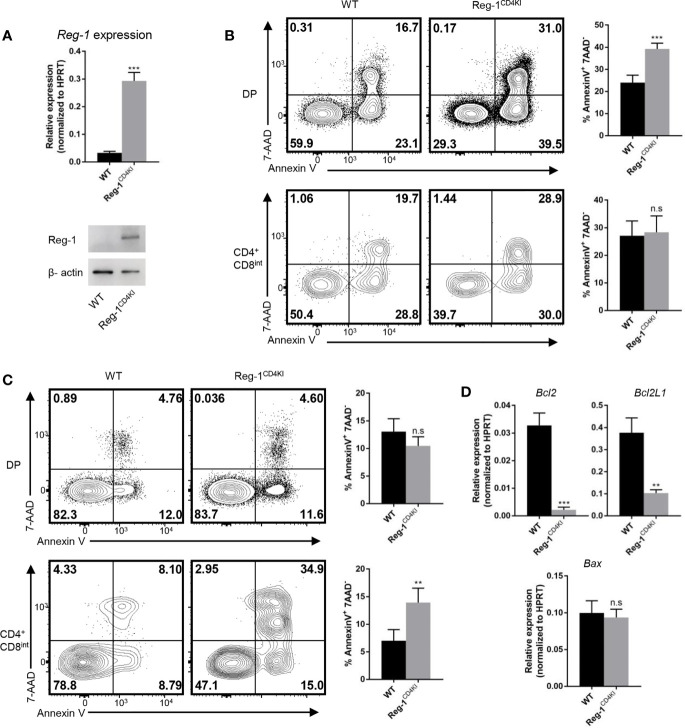
Thymocytes that express mutant Regnase-1 are more susceptible to apoptosis. **(A)** Immunoblot analysis of Regnase-1 expression and RT-qPCR analysis of Regnase-1 mRNA in sorted DP thymocytes. **(B)** Flow cytometric analysis of the apoptosis of DP and CD4^+^CD8^int^ thymocytes with anti-CD3 (5 µg/ml) and anti-CD28 (1 µg/ml) for 24h. Bar graph indicates Annexin V^+^7-AAD^-^ fraction of DP and CD4^+^CD8^int^ thymocytes. **(C)** Flow cytometric analysis of the apoptosis of DP and CD4^+^CD8^int^ thymocytes with PMA (50 ng/ml) and ionomycin (500 ng/ml) stimulation for 4h. Bar graph indicates Annexin V^+^7-AAD^-^ fraction of DP and CD4^+^CD8^int^ thymocytes. **(D)** RT-qPCR analysis of apoptotic genes expression. Data are mean ± SD (n=3) from one experiment, representative of three independent experiments. n.s not significant; **P < 0.01; ***P < 0.001 (unpaired two-tailed Student’s *t* test).

We further investigated the influence of mutant Regnase-1 on thymocyte apoptosis. We sorted DP thymocytes and detected the mRNA and protein of Regnase-1. We confirmed that the mutant Regnase-1 was expressed in DP cells from Reg-1^CD4KI^ mice (**Figure 7A**). We treated thymocytes with anti-CD3 and anti-CD28 to stimulate TCR on thymocytes, and more DP thymocytes in Reg-1^CD4KI^ mice were undergoing apoptosis (**Figure 7B**). However, cell viability of DP thymocytes was not affected in the absence of TCR stimulation (**Supplementary Figure 4A**), indicating the susceptibility of DP T cells to apoptosis was due to the inappropriate TCR signaling. In addition, PMA and ionomycin, the nonspecific agonists to stimulate mature T cells, could lead to more apoptosis of CD4^+^CD8^int^ thymocytes in Reg-1^CD4KI^ mice (**Figure 7C**). For DN thymocytes, cell viability was not influenced (**Supplementary Figures 4B, C**). Regnase-1 is known as an RNase that destabilizes a set of mRNAs (1, 24). We stimulated DP thymocytes with anti-CD3 and anti-CD28 for 3h and detected the expression of apoptosis-related genes. We found that the mRNA of anti-apoptotic genes *Bcl-2* and *Bcl2L1* were significantly lower in Regnase-1 knock-in DP thymocytes, but the level of pro-apoptotic gene *Bax* mRNA was comparable (**Figure 7D**). Taken together, these data suggested the mutant Regnase-1 could downregulate the expression of anti-apoptotic genes in thymocytes, which resulted in their apoptosis in thymus.

## Discussion

Regnase-1 is an RNA binding protein with potent endoribonuclease activities and traditionally thought to play a vital role in regulating mRNA stability (2, 25). In the present study, we created a Regnase-1 mutant and expressed this mutant *in vivo* as a transgene in an attempt to examine the impact of Regnase-1 on the T cell response. We found that the Regnase-1 mutant transgenic mice displayed profound lymphopenia, with few mature T cells in the periphery. Among the T cells in the periphery, however, most showed an effector memory phenotype, as they downregulated CD62L and expressed high CD44. Interestingly, those mutant mice failed to reject skin allografts, a response that depends on T cells, suggesting that such peripheral effector memory T cells are not sufficient to reject allografts. In fact, those peripheral T cells express much reduced levels of the effector cytokines TNF-α and IFN-γ. Mechanistically, the mutant Regnase-1 impaired T cell thymic positive selection, most likely *via* its inhibitory effect on TCR-β expression and TCR signaling. As a consequence, thymocytes that expressed mutant Regnase-1 were susceptible to apoptosis due to the lack of TCR-triggered survival signal.

In our transgenic mice, we replaced the arginine 111 of Regnase-1 with alanine to improve its stability *in vivo*. Our data suggested that R111A mutant Regnase-1 was resistant to Malt1 mediated cleavage without interfering with its endoribonuclease activities. Mino, T. et al. reported that Regnase-1 fails to degrade mRNAs in the absence of UPF1 (4). In our luciferase assay, the R111A mutant Regnase-1 had normal Rnase activity, indicating that the interaction between the R111A mutant Regnase-1 and UPF1 was not affected. A number of studies have shown that Regnase-1 is uniquely important in controlling T cell activation. For example, in Regnase-1 deficient mice, T cells are spontaneously activated, resulting in fetal autoimmune disease (8). In our transgenic mice, the mutant Regnase-1 led to fewer peripheral CD4^+^ and CD8^+^ T cells and Tregs in spleen, supporting an inhibitory role of Regnase-1 in T cell activities. In lymphopenic mice, residual T cells usually undergo robust homeostatic expansion, thus giving rise to population of T cells with an effector memory phenotype (CD44^hi^CD62L^lo^) (23). We observed that in the Reg-1^CD4KI^ mice, such effector memory T cells are functionally impaired, as they completely failed to expand and reject the skin allografts. The rejection to allografts depends on the number and function of alloreactive T cells (26, 27). Although with the stimulation of alloantigens, the peripheral T cells in transgenic mice could express activation markers (ICOS, OX40, PD-1, KLRG1), but expressed reduced levels of cytokines (IFN-γ, TNF-α). The expansion of alloreactive effect T cells was inhibited in transgenic mice. Thus, the reduced number of T cells in the periphery and the impaired functions of peripheral T cells may both contribute to the acceptance of skin allografts. In this study, at 10 days post skin transplantation, fewer effect T cells infiltrated into the skin allograft in transgenic recipients, indicating the reduced anti-graft T cells. Although the peripheral T cells showed reduced effector functions, approaches to selectively overexpress mutant Regnase-1 in mature T cell may yield additional insights into the regulatory role of Regnase-1 in T effector cell activities.

Our finding that in the thymus of transgenic mice, the inhibition of TCR expression and/or signaling at the DP stage by mutant Regnase-1 was highly interesting. Previous studies showed that the deletion of Regnase-1 or expression of protease-dead Malt1 in T cells has no obvious effects on T cell development (8, 28), which is very different from our findings, highlighting the complexity of Regnase-1 in regulating T cell biology. Clearly, the interaction of TCR with self-peptide/MHC complex is vital for the fate of developing T cells. For example, in *Rag* ^-/-^ mice, the lack of antigen receptor leads to the loss of all mature lymphocytes (29, 30). In our transgenic mice, the mutant Regnase-1 was initially expressed in DP thymocytes under the control of CAG promoter, enabled by *Cd4*-Cre mediated excision of the stop cassette. Although the exact mechanisms remain to be defined, a lower frequency of post-selection thymocytes (TCR^hi^CD69^hi^) indicated a defect of thymic positive selection in Regnase-1 mutant mice, most likely due to an impaired TCR-β expression and signaling. Also, CD69 can directly compete with S1P1, a chemokine receptor that is required for mature thymocyte egression from thymus (31–33). Developing thymocytes with high level of CD69 can be retained in the thymus until their maturation (34). Thus, the DP thymocytes from Reg-1^CD4KI^ mice might egress from thymus, and the lack of interaction with thymic stromal cells could impair thymic selection. In addition, the upregulation of CD5 during thymocytes development may be blocked by mutant Regnase-1, indicating its profound suppressive effect on TCR signaling. Overexpression of Regnase-1 affected translocation of TCR-β from cytoplasm to membrane, which can also contribute to the insufficient of TCR signal during T cell development. TCR signaling provides survival signal for T cells during thymic selection, and Regnase-1 has been reported to enhance mRNA decay of several anti-apoptotic genes including *Bcl2L1, Bcl2A1, RelB, Birc3*, and *Bcl3* (24). We provided evidence that the mRNA of anti-apoptotic genes (*Bcl-2*, *Bcl2L1*) were significantly lower in Regnase-1 knock-in DP T cells, and these T cells were more susceptible to apoptosis with TCR stimulation.

Our study suggests that the ribonuclease Regnase-1 could be therapeutically targeted to modulate T cell activities under various circumstances, including transplant survival. But the clinical implications of our approach remain uncertain. Clearly, the induction of profound lymphopenia is not ideal and would expose patients to greater risks of immunodeficiency. However, the finding that the Regnase-1 mutant exhibits an inhibitory effect in TCR signaling and effector activities may open the door in the development of novel therapeutic approaches in treatment of multiple T cell-mediated immune diseases.

## Data Availability Statement

The original contributions presented in the study are included in the article/**Supplementary Material**. Further inquiries can be directed to the corresponding author.

## Ethics Statement

The animal study was reviewed and approved by Houston Methodist Animal Care Committee.

## Author Contributions

GK, YD, XX, and XL were involved in the design of the study. GK, YD, and YW performed the experiments and analysed the data. GK, YM, and XL contributed to the writing and editing of the manuscript. All authors contributed to the article and approved the submitted version.

## Funding

This work was supported by grant from National Institutes of Health (R01AI129906) and the Methodist startup award.

## Conflict of Interest

The authors declare that the research was conducted in the absence of any commercial or financial relationships that could be construed as a potential conflict of interest.
