# Supplementary material for: Transgenic Expression of a Mutant Ribonuclease Regnase-1 in T Cells Disturbs T Cell Development and Functions
Source: Front Immunol. 2021 Jul 8;12:682220. doi: 10.3389/fimmu.2021.682220 (PMC8297167; doi:10.3389/fimmu.2021.682220)

Supplementary Material

Supplementary figure 1. (A) Cross *Reg-1*KI mice with *Cd4*cre mice to conditional overexpress R111A mutant Regnase-1 in T cells. (B) Genotyping image of Reg-1^{CD4KI} and their WT littermate. (C) Gross appearance of thymus and spleen from WT or Reg-1^{CD4KI} mice.

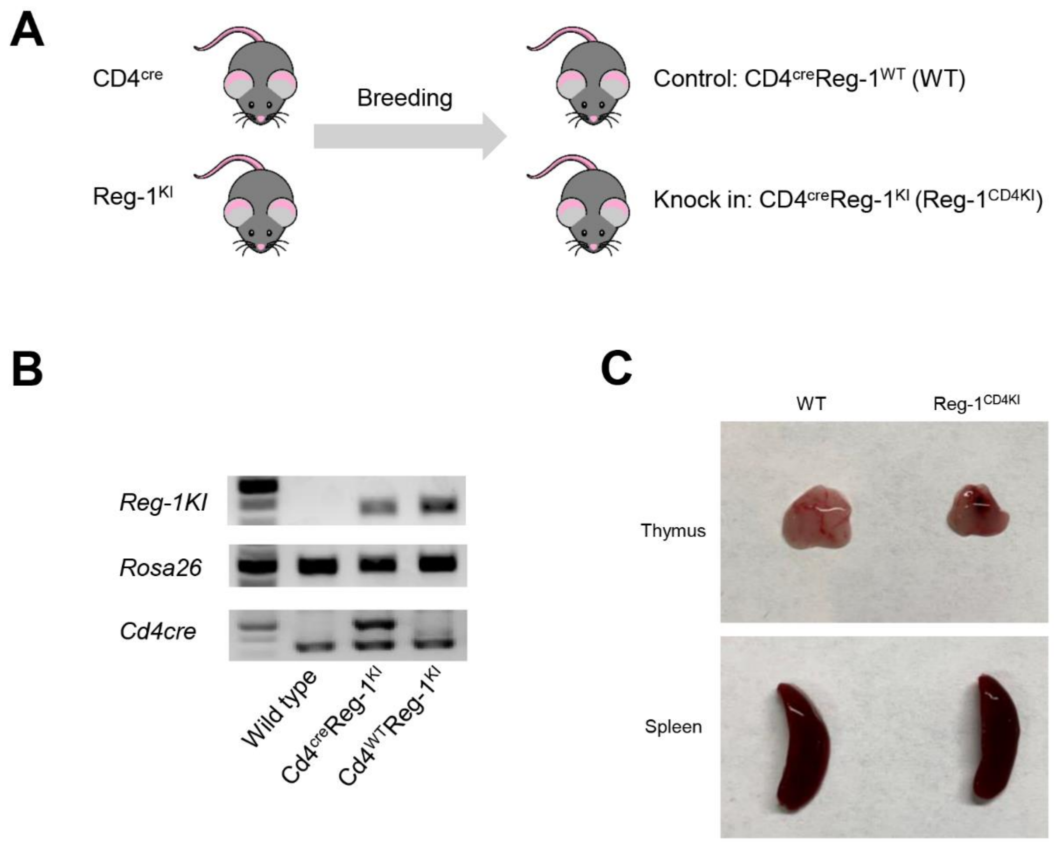

Supplementary figure 2. (A) The fraction of Treg in spleen. (B) At day 10 post skin transplantation, flow cytometry analysis of activation markers on splenic $CD44^+$ T cells. Data are mean ± SD (n=3) from one experiment, representative of three independent experiments. n.s not significant; ***P*<0.01 (unpaired two-tailed Student's *t* test).

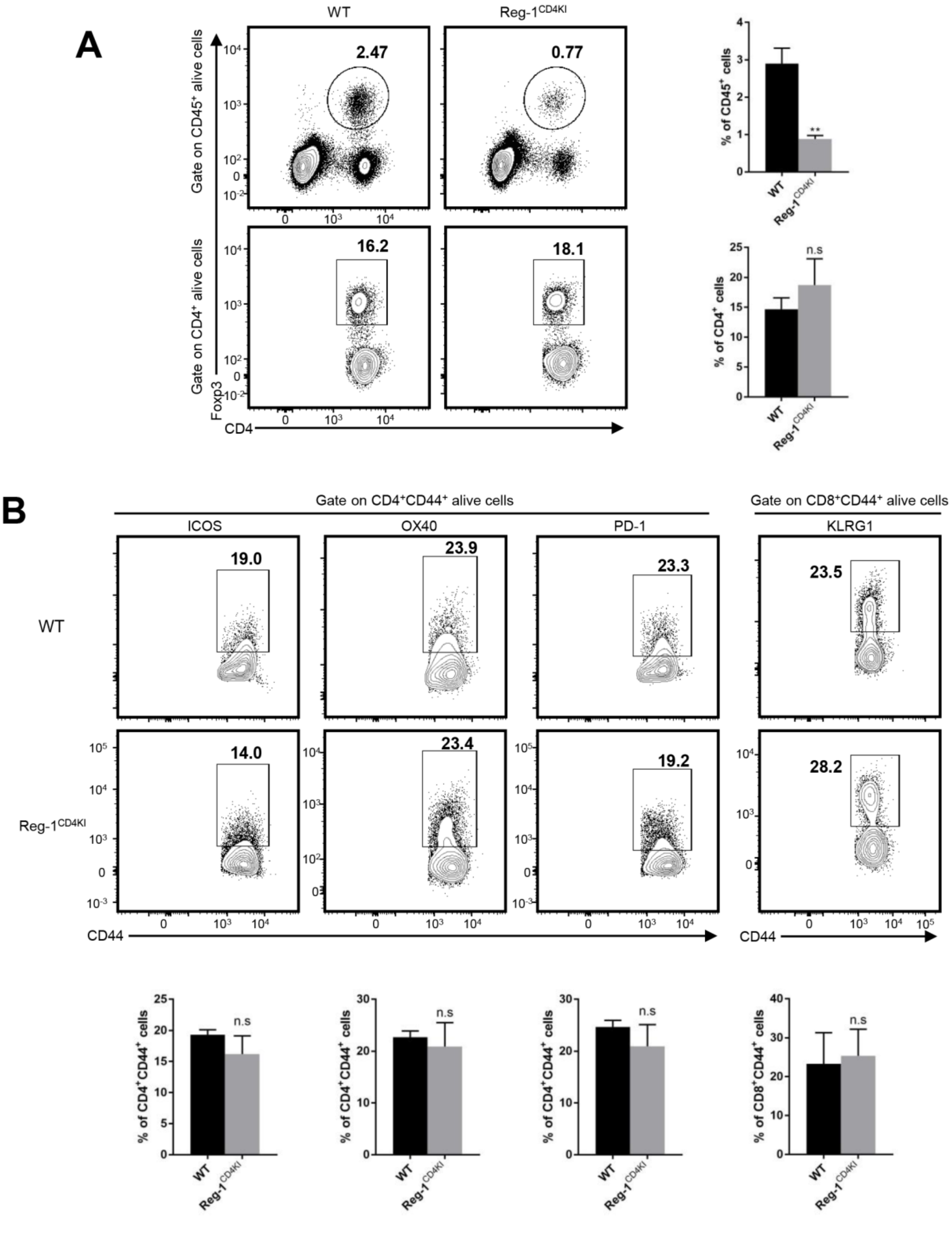

Supplementary figure 3. (A) Flow cytometric analysis of DN1 to DN4 cell development based on the expression of CD44 and CD25, gated on $CD45^{+}CD4^{-}CD8^{-}$ alive thymocytes. (B) Surface staining of CD4 and CD8 on thymocytes at population 1-5. Numbers above the plots indicate five subpopulations based on the expression level of CD69 and TCR-β, and numbers in parentheses indicate percent thymocytes in each subpopulation. (C) Intracellular and surface staining of TCR-β on DN, DP and CD4SP thymocytes from WT and Reg-1^{CD4KI} mice. Data are mean ± SD (n=5) from one experiment, representative of three independent experiments. n.s not significant; *** P<0.001 (unpaired two-tailed Student's t test).

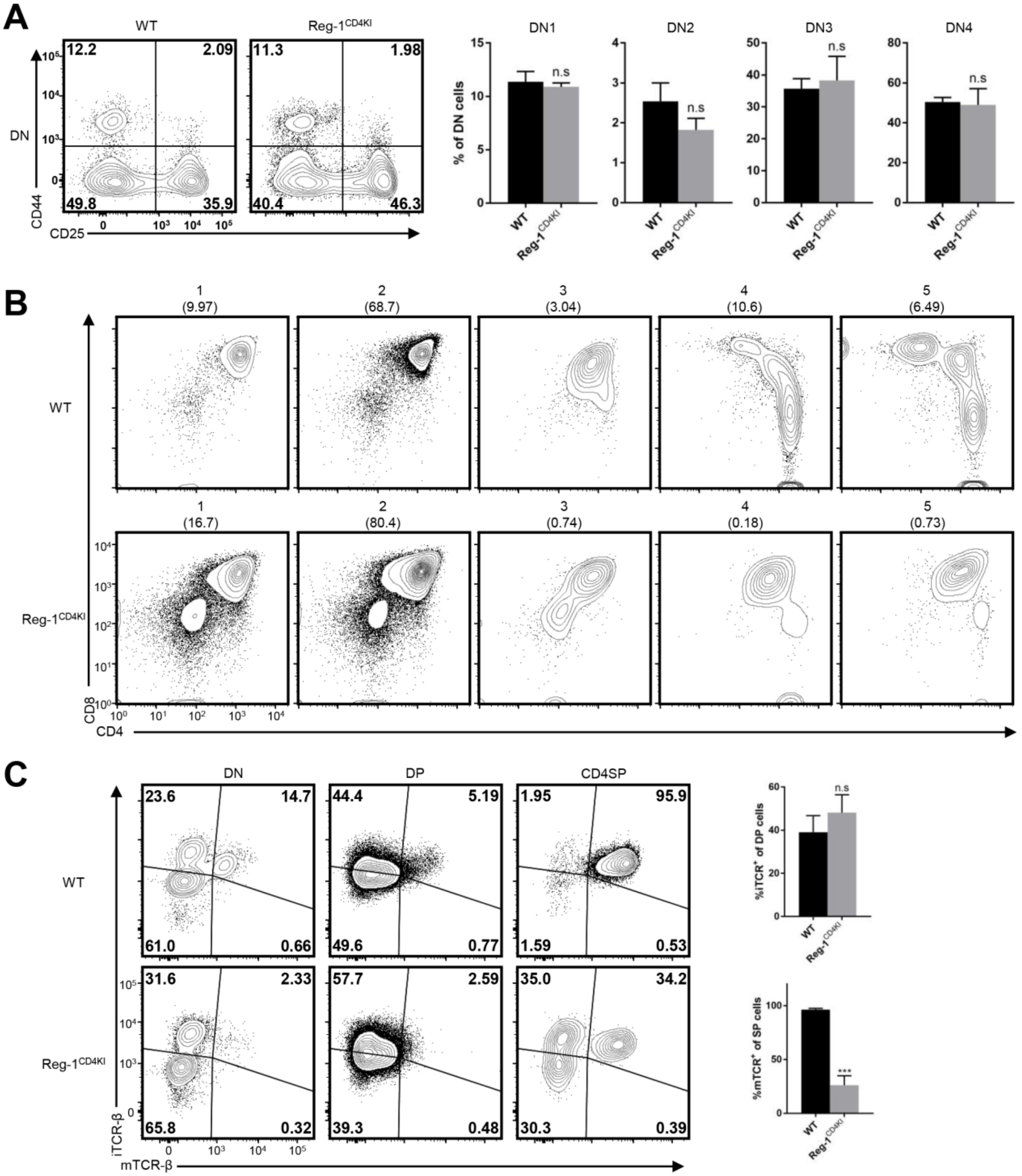

Supplementary figure 4. (A) Flow cytometric analysis of the apoptosis of DP thymocytes without anti-CD3 and anti-CD28 stimulation for 24h. (B) Flow cytometric analysis of the apoptosis of DN thymocytes with anti-CD3 (5 µg/ml) and anti-CD28 (1 µg/ml) for 24h. (C) Flow cytometric analysis of the apoptosis of DN thymocytes with PMA (50 ng/ml) and ionomycin (500 ng/ml) stimulation for 4h. Data are mean ± SD (n=3) from one experiment, representative of two to three independent experiments. n.s not significant; **P*<0.05 (unpaired two-tailed Student's *t* test).

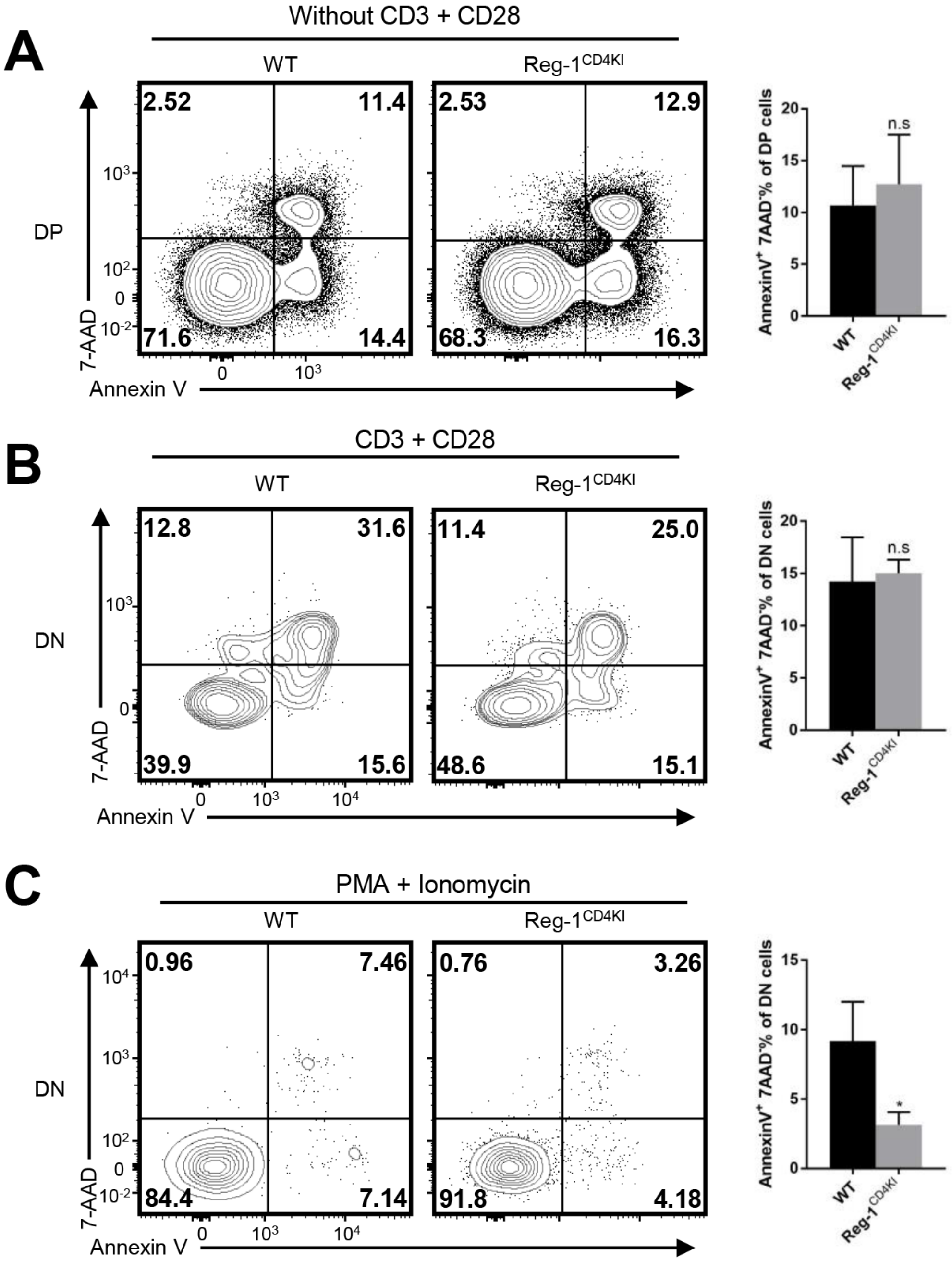

Supplement: Supplementary file 1 [file DataSheet_1.pdf]
