# Supplementary material for: Transgenic Expression of a Mutant Ribonuclease Regnase-1 in T Cells Disturbs T Cell Development and Functions
Source: Front Immunol. 2021 Jul 8;12:682220. doi: 10.3389/fimmu.2021.682220 (PMC8297167; doi:10.3389/fimmu.2021.682220)
Supplement: Supplementary file 2 [file Table_1.docx]

Supplementary Material

Supplementary table 1. List of primers used for RT-qPCR

|  |  | Primer |
| --- | --- | --- |
| HPRT | Forward | 5'- AGTACAGCCCCAAAATGGTTA -3' |
|  | Reverse | 5'- CTTAGGCTTTGTATTTGGCTTT -3' |
| Regnase-1 | Forward | 5'- AGATATTACCGTGTTTGTGC -3' |
|  | Reverse | 5'- CTTTTTCTCTAGTTCCCGAAG -3' |
| Bcl-2 | Forward | 5'- GTCGCTACCGTCGTGACTTC -3' |
|  | Reverse | 5'- CAGACATGCACCTACCCAGC -3' |
| Bcl2L1 | Forward | 5'- GACAAGGAGATGCAGGTATTGG -3' |
|  | Reverse | 5'- TCCCGTAGAGATCCACAAAAGT -3' |
| Bax | Forward | 5'- TGAAGACAGGGGCCTTTTTG -3' |
|  | Reverse | 5'- AATTCGCCGGAGACACTCG -3' |
